# Role of Mitogen-Activated Protein Kinases in Myocardial Ischemia-Reperfusion Injury during Heart Transplantation

**DOI:** 10.1155/2012/928954

**Published:** 2012-03-18

**Authors:** Giuseppe Vassalli, Giuseppina Milano, Tiziano Moccetti

**Affiliations:** ^1^Fondazione CardioCentro Ticino, Via Tesserete, 6900 Lugano, Switzerland; ^2^Department of Cardiology, University of Lausanne Medical Center, Av. du Bugnon, 1011 Lausanne, Switzerland; ^3^Department of Cardiovascular Surgery, University of Lausanne Medical Center, Av. du Bugnon, 1011 Lausanne, Switzerland

## Abstract

In solid organ transplantation, ischemia/reperfusion (IR) injury during organ procurement, storage and reperfusion is an unavoidable detrimental event for the graft, as it amplifies graft inflammation and rejection. Intracellular mitogen-activated protein kinase (MAPK) signaling pathways regulate inflammation and cell survival during IR injury. The four best-characterized MAPK subfamilies are the c-Jun NH2-terminal kinase (JNK), extracellular signal- regulated kinase-1/2 (ERK1/2), p38 MAPK, and big MAPK-1 (BMK1/ERK5). Here, we review the role of MAPK activation during myocardial IR injury as it occurs during heart transplantation. Most of our current knowledge regarding MAPK activation and cardioprotection comes from studies of preconditioning and postconditioning in nontransplanted hearts. JNK and p38 MAPK activation contributes to myocardial IR injury after prolonged hypothermic storage. p38 MAPK inhibition improves cardiac function after cold storage, rewarming and reperfusion. Small-molecule p38 MAPK inhibitors have been tested clinically in patients with chronic inflammatory diseases, but not in transplanted patients, so far. Organ transplantation offers the opportunity of starting a preconditioning treatment before organ procurement or during cold storage, thus modulating early events in IR injury. Future studies will need to evaluate combined strategies including p38 MAPK and/or JNK inhibition, ERK1/2 activation, pre- or postconditioning protocols, new storage solutions, and gentle reperfusion.

## 1. Introduction

Heart transplantation is the final therapeutic option for heart failure [1]. Over the past two decades, advances in immunosuppression and antimicrobial agents have improved outcomes after heart transplantation. An analysis of the UNOS database in 14,401 first-time orthotopic heart transplant recipients between the years 1999 and 2006 showed that the survival rate at 30 days, 1 year, and 5 years was 94%, 87%, and 75%, respectively, for the young group (<60 years of age) and 93%, 84%, and 69% for the older group [2]. Graft vasculopathy, a unique form of accelerated coronary artery disease, is a major cause of late graft failure [3]. The disease is characterized by intimal thickening mainly due to smooth muscle cell proliferation and fibrosis. Occlusive narrowing of the coronary vessels can develop within a few months and is not prevented by current treatments.

The pathogenesis of graft vasculopathy is complex and has been reviewed elsewhere [4–6]. The observation that, while graft coronary arteries develop lesions, the host's native arteries are spared suggests a major pathogenic role for immune rejection. Consistent with this, while hearts transplanted into a genetically different recipient are affected, those placed back in the original donor strain are spared [7]. Clinical data support a major role for chronic rejection in the development of graft vasculopathy and graft failure. Indeed, the degree of donor-recipient human leukocyte antigen (HLA) matching correlates significantly with graft survival [8–10]. Moreover, acute cellular rejection has been associated with an increased risk of developing graft vasculopathy [11–14].

Both the innate [15] and the adaptive immune system including B cells and antibody formation against graft antigens [16] play central roles in the development of graft vasculopathy. Nonimmunological factors such as dyslipidemia, hypertension, drug toxicity, and infections also play contributory roles. Accordingly, the current paradigm is that graft vasculopathy results from repeated immune and nonimmune-mediated insults to graft coronary endothelium leading to endothelial inflammation and dysfunction, vascular cell proliferation, fibrosis, and intimal thickening.

Extended cold ischemic times during heart transplantation have been associated with increased risk of developing graft vasculopathy and failure both in animal models [17, 18] and in humans [19]. Moreover, prolonged times between donor brain death and organ retrieval have been associated with increased mortality in cardiac transplant recipients [20]. Graft coronary microvascular dysfunction after ischemia and reperfusion can culminate in primary graft failure or untreatable chronic rejection [21]. 

Cold ischemia stimulates the expression of inflammatory mediators acting as “danger signals” and amplifying tissue injury and graft rejection. Toll-like receptors (TLRs) play a central role in this regard [22]. Consistent with this, systemic administration of anti-TLR-2 antibody reduces neutrophil, macrophage, and T-lymphocyte infiltration in mouse hearts after ischemia and reperfusion [23]. Multiple strategies applied at the time of organ transplantation have a potential for limiting cold ischemic organ damage, reperfusion injury, and graft immunogenicity [24, 25]. 

## 2. Myocardial Ischemia/Reperfusion (IR) Injury

Early observations in animal models of myocardial infarction indicated that ischemic cell death progresses as a “wavefront” phenomenon correlated to the duration of ischemia [26], and that early reperfusion can salvage reversibly injured ischemic myocardium [27]. Subsequently, morphological changes appearing during reperfusion, including cardiomyocyte swelling and loss of sarcomeric organization, were recognized [28]. Moreover, interventions applied at the onset of reperfusion were still able to limit infarct size, suggesting a contributory role for reperfusion in lethal cell injury.

A comprehensive discussion of the molecular mechanisms of myocardial IR injury is beyond the scope of the present paper. These mechanisms have been reviewed elsewhere [29, 30]. It is possible here to briefly mention the role of mitochondria as both a source and a target of IR injury [31, 32]. Under normoxic conditions, mitochondria use oxygen to synthesize adenosine triphosphate (ATP). Sustained hypoxia leads to ATP depletion, acidosis, intracellular calcium accumulation, mitochondrial swelling, and cell death [30]. Cold ischemia exacerbates swelling via inhibition of the Na^+^/K^+^ ATPase. At reperfusion, calcium is taken up into the sarcoplasmic reticulum (SR) by the SR calcium ATPase. Calcium overload then leads to calcium release into the cytosol, cardiomyocyte hypercontracture, membrane disruption, and cell death [30]. 

During ischemia, mitochondria produce reactive oxygen species (ROS). An extra burst of ROS generation takes place at reperfusion. ROS mediates opening of the mitochondrial permeability transition pore (MPTP) leading to increased inner mitochondrial membrane permeability, mitochondrial depolarization, ATP depletion, mitochondrial matrix swelling, outer mitochondrial membrane rupture, cytochrome *c* release, and apoptosis [30, 32]. In addition, ROS activates multiple molecular cascades of inflammation [33]. Proinflammatory cytokines, such as IL-1 and TNF*α*, and chemokines are produced within hours of reperfusion in allogeneic and syngeneic grafts alike. Chemokines mediate early migration of neutrophils and macrophages into the graft [34, 35]. Early T-cell reaction precedes alloantigen priming and induces graft necrosis [36, 37]. Inflammatory activation of graft endothelium [38], platelets, the coagulation cascade, and the complement system [39] plays important roles in early graft injury and subsequent graft vasculopathy.

A multitude of intracellular signal transduction pathways are activated during myocardial IR injury [29, 30]. Among them, mitogen-activated protein kinases (MAPKs) are key regulators of cell function and survival [40, 41]. The present paper aims to discuss the role of MAPK activation in myocardial IR injury and its potential implications for heart transplantation.

## 3. MAPK Subfamilies

**Figure 1:**
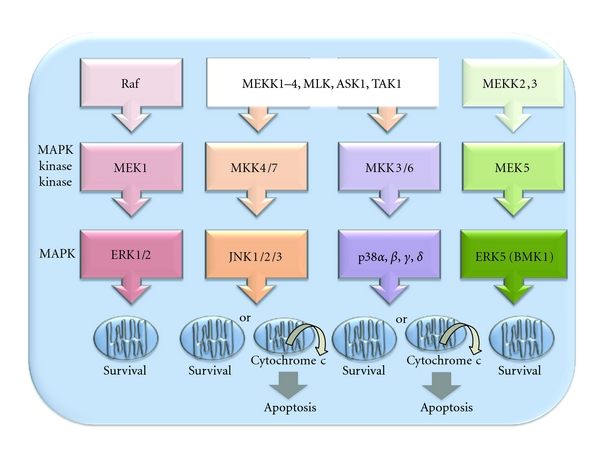
Schematic depicting the activation cascades of the four major MAPK subfamilies and corresponding effects on mitochondrial chromosome *c* release and apoptosis. ERK1/2 and ERK5/BMK1 have been associated with cell survival, whereas JNK and p38 MAPK have been predominantly associated with apoptosis.

The MAPK family includes four major serine/threonine protein kinase subfamilies. Each MAPK subfamily comprises successively acting kinases including an upstream MAPK kinase kinase, a MAPK kinase, and a MAPK (Figure 1) [40]. Distinct isoforms of a MAPK bind molecules with different affinities and can activate distinct signaling pathways. In response to a variety of stress stimuli, MAPKs convey extracellular signals to their intracellular targets, thereby regulating cell survival, function, growth, and differentiation [41]. The best characterized MAPK subfamilies are c-Jun NH_2_-terminal kinases (JNKs), extracellular signal-regulated kinase-1/2 (ERK1/2, also known as p42/p44 MAPK), p38 MAPKs, and the big MAPK-1 (BMK1/ERK5). The role of each MAPK subfamily in myocardial IR injury is discussed in the next sections.

### 3.1. ERK1/2 Activation during Myocardial IR Injury

ERK1/2 was discovered as the first member of the MAPK family in 1990 [42]. This serine/threonine protein kinase is tyrosine-phosphorylated in response to various extracellular signals. We observed a *≈*2-fold increase in ERK1/2-specific in vitro kinase activity in isolated-perfused adult rat hearts subjected to 20 min of ischemia followed by 15 min of reperfusion [43]. Several studies support a protective role for the MEK1-ERK2 signaling pathway against IR injury [44–47]. Accordingly, this pathway has been identified as a central component of the so-called “Reperfusion Injury Salvage Kinase” (RISK) pathway [48].

### 3.2. JNK Activation during Myocardial IR Injury

JNK was discovered as the second member of the MAPK family in 1991 [49]. It is primarily activated by various cellular stresses such as heat, UV light, and cytokines. We observed a *≈*6-fold increase in JNK-specific in vitro kinase activity and a *≈*2-fold increase in phosphorylated c-Jun protein in nuclear extracts from isolated-perfused rat hearts subjected to 20 min of ischemia and 15 min of reperfusion [43]. JNK activation was increased during ischemia as well as reperfusion, in line with a limited number of previous studies [44, 50, 51]. In contrast, a larger number of studies reported JNK activation predominantly at reperfusion [52–56]. 

Dichotomous effects of JNK activation during IR injury including both cardioprotection [56–59] and myocardial damage [55, 60–64] have been reported. A potential mechanism of JNK-mediated protection is reactivation of Akt and enhanced cardiomyocyte survival after hypoxic injury [56]. Data in genetically modified mice show that JNK1/2 knockout mice and, paradoxically, transgenic mice overexpressing MKK7, the MAPK kinase upstream of JNK1/2, are each significantly protected from IR injury [65]. These findings illustrate the complexity of the biological effects of JNK activation.

A word of caution is warranted regarding the reliance on curcumin as a specific JNK inhibitor in early studies [66]. We therefore used a cell-penetrating peptide inhibitor of JNK, D-JNKI-1, as a more selective agent. In the isolated-perfused adult rat heart, D-JNKI-1 administered before the ischemic period selectively prevented JNK activation and improved post-ischemic cardiac function, cytochrome *c* release, caspase-3 activation, and apoptosis [43]. D-JNKI-1 administered at reperfusion failed to improve cardiac function but still prevented apoptosis. In vivo, D-JNKI-1 reduced myocardial infarct size by half after coronary artery occlusion and reperfusion in rats [43]. D-JNKI-1 similarly reduced cerebral infarct size after common carotid artery occlusion and reperfusion in adult rats [67]. 

Inconsistent findings from previous studies regarding the role of JNK activation during IR injury likely reflect differences in the experimental models and JNK inhibitors used, as well as JNK isoform-specific effects. It has been shown that inhibition of JNK1 isoform, but not of JNK2 isoform, prevents apoptosis induced by IR injury in rat cardiomyocytes [61]. 

### 3.3. p38 MAPK Activation during Myocardial IR Injury

The p38 MAPK subfamily comprises 4 main isoforms, p38*α*, p38*β*, p38*γ*, and p38*δ*, of which p38*α* and p38*γ* are most abundantly expressed within the myocardium. The role of p38 MAPK activation during myocardial IR remains controversial [68–70]. We observed a *≈*2-fold increase in p38 MAPK-specific in vitro kinase activity in isolated-perfused rat hearts subjected to 20 min of ischemia and 15 min of reperfusion [43]. These results are in agreement with previous data [71]. p38 MAPK activation contributes to tissue injury induced by TNF*α* in response to hydrogen peroxide generated during reperfusion [33]. Moreover, p38 MAPK activation counteracts adenosine- or insulin-induced cardioprotection against IR injury [72, 73]. p38 MAPK inhibition limits infarct size and polymorphonuclear accumulation in mouse hearts subjected to IR injury [74]. Transgenic mice expressing a dominant-negative p38*α* mutant or a dominant-negative mutant of MKK6, a MAPK kinase upstream of p38 MAPK, are each significantly protected from IR injury [75]. These data suggest a potential role for p38*α* isoform as a mediator of myocardial IR injury.

Much of our current knowledge regarding cardioprotection comes from studies of preconditioning (PC) and postconditioning (PostC). Although a majority of these studies relate to nontransplanted hearts, they are relevant to heart transplantation.

## 4. Ischemic Preconditioning (IPC)

IPC was originally described as an experimental phenomenon whereby repeated episodes of brief, sublethal ischemia induced tolerance to a successive, prolonged period of lethal ischemia [30, 76, 77]. In the anesthetized dog, four 5 min periods of occlusion of the left coronary artery, interspersed with 5 min periods of rapid reflow, markedly attenuated infarct size after occlusion of the same artery for 40 min. Two distinct “windows” of IPC-mediated protection have been described [78, 79]. The first window of protection is induced within minutes, lasts for 1-2 h, is dependent on activation of MAPKs as well as of other signaling pathways, and attenuates infarct size but not contractile dysfunction nor myocardial stunning. The second window of protection takes place between 24 and 72 h after the triggering phase of IPC, requires synthesis of protective proteins within the heart, and limits cell death as well as contractile dysfunction [80]. IPC involves changes in energy metabolism, ionic homeostasis, and gene regulation as well as a decrease in ROS generation, neutrophil activation, and apoptosis [81]. Pharmacological agents such as opioids [82], inhalational anesthetics [83], adenosine, isoproterenol, and nitric oxide (NO) donors, [84] along with stress stimuli such as rapid cardiac pacing and thermal stress can precondition myocardial tissue to subsequent ischemia [30]. 

A comprehensive discussion of the molecular mechanisms of IPC is beyond the scope of the present paper. The interested reader is referred to recent reviews published elsewhere [30, 77, 85, 86]. It is possible here to merely mention a few molecular mechanisms. While the triggering phase of IPC requires NO and superoxide synthesis, IPC mitigates NO, superoxide, and peroxynitrite overproduction during subsequent IR [87]. Beside MAPKs, protein kinases activated by IPC include protein kinase C (PKC) isoforms [88, 89], phosphatidylinositol 3-kinase (PI3K) and its substrate kinase Akt [90, 91], receptor tyrosine kinases of the Src family [92, 93], the JAK/STAT pathway [94, 95], and glycogen synthase-3*β* (GSK-3*β*) [96]. The latter is a downstream kinase phosphorylated by other kinases such as ERK1/2 and Akt which has been implicated in cardioprotection including inhibition of MPTP opening at reperfusion. However, recent data suggest that decreased oxidative stress, rather than mitochondrial protein phosphorylation, is responsible for inhibition of MPTP opening in the context of IPC [97]. 

A number of studies have demonstrated MAPK activation during the triggering phase of IPC, at reperfusion, or both. In some cases, IPC has been associated with decreased MAPK activation during subsequent ischemia, suggesting a detrimental role for MAPK activation in this context. The activation of the different MAPK subfamilies in preconditioned hearts is discussed in the next sections.

### 4.1. ERK1/2 Activation during IPC

Both in vitro and in vivo studies have demonstrated ERK1/2 activation and cardioprotection after IPC [98–101], which was abolished by an ERK1/2 inhibitor in a pig model of IR injury [99]. In addition, hypoxic PC [102, 103] as well as delayed hypoxic PC [104, 105], adenosine-induced PC [106] as well as adenosine-induced delayed PC [107], isoflurane/desflurane-induced PC [83, 108], metabolic PC [109], and opioid-induced delayed PC are associated with increased ERK1/2 activation [110]. Moreover, mitochondrial K_ATP_ channel openers activate ERK1/2 by an oxidant-dependent mechanism [111]. 

Several studies reported biphasic ERK1/2 activation during IPC [82, 83]. The first phase of activation takes place immediately after the PC stimulus, and the second phase of activation occurs at reperfusion. Blocking the first phase of activation prevents the second one [83]. In response to IPC, PKC*ε* induces the activation of ERK1/2 in the cytosol and its translocation to the nucleus, with increased activation of NF-kB and AP-1 transcription factors and protection against cardiomyocyte apoptosis [101]. Another mechanism by which ERK1/2 can impart protection to hypoxic myocardium involves phosphorylation of hypoxia-inducible factor (HIF)-1 [104]. 

A small number of studies either reported ERK1/2 activation during IPC [90] or metabolic preconditioning [112] without a contribution of it to the observed protection, or failed to detect ERK1/2 activation during IPC [113, 114]. 

### 4.2. JNK Activation during IPC

Several studies documented increased JNK activation during the triggering phase of IPC [55, 98, 100, 101, 113, 115–117] or, less frequently, during the sustained ischemic period after the IPC stimulus [113] or during reperfusion [55, 117]. Some studies suggested a potential role for JNK as a mediator of IPC-induced protection [100, 116], but this was not confirmed by other reports [117, 118]. Decreased JNK activation was observed in preconditioned brains, kidneys, and hepatocytes [119–121], suggesting that JNK activation may contribute to IR injury in these tissues.

### 4.3. p38 MAPK Activation during IPC

Several studies reported increased p38 MAPK activation during the triggering phase of IPC and reperfusion [83, 113, 116, 117, 122–132]. A limited number of studies showed p38 MAPK activation during the sustained ischemic period after the IPC stimulus [133–135]. p38 MAPK activation has also been observed in hypoxic PC [136, 137] and delayed hypoxic PC [138] as well as in NO, [139], angiotensin II [140], or adenosine-induced PC [115, 141–143]. 

The role of p38 MAPK as a potential mediator of protection in the preconditioned heart remains controversial. A majority of studies showed p38 MAPK activation during the triggering phase of PC [85, 110, 116, 122, 123, 125, 127–129, 131–138, 140, 141, 143–145]. IPC appears to require p38*α* but not p38*β* isoform activation [145]. Potential p38 MAPK-mediated protective mechanisms include phosphorylation of small heat shock protein (Hsp) 27, which stabilizes the actin cytoskeleton [146–148], and *αβ* crystalline [124]. 

A distinct group of studies failed to support a contributory role for p38 MAPK activation in IPC [50, 110, 149–152], hypoxic PC [140, 153], NO-induced PC [154], delayed metabolic PC [109, 112], and opioid-induced delayed PC [110]. A third group of studies showed reduced p38 MAPK activation during the sustained ischemic period after the PC stimulus [140, 150, 152–154], suggesting a detrimental role for p38 MAPK activation in this setting. Consistent with this, numerous studies demonstrated that a p38 MAPK inhibitor applied during the sustained ischemic period can protect the myocardium against IR injury [44, 116, 125, 126, 140, 147, 149–153, 155, 156]. 

These inconsistent findings from different studies are difficult to reconcile; however, it should be considered that the mechanism of p38 MAPK activation can differ by circumstance [70], and that distinct p38 MAPK isoforms activate different signaling pathways. Increased p38*α* isoform activation during sustained ischemia [50, 153] has been associated with cardiomyocyte apoptosis [157, 158], contractile dysfunction [158], and increased infarct size [159]. p38 MAPK has been shown to negatively regulate myocardial contractility [160–162]. 

### 4.4. IPC and BMK1/ERK5 Activation

The big MAP kinase 1 (BMK1/ERK5) pathway [163] is activated in the heart in response to IPC [164] and has been implicated as a potential mediator of cardioprotection [165]. BMK1/ERK5-induced phosphorylation of the mitochondrial protein BAD has been shown to attenuate endothelial cell and cardiomyocyte apoptosis [166–168]. Similarly, BMK1/ERK5 activation during cerebral IPC prevents apoptosis in the ischemic rat hippocampal CA1 region [169]. 

## 5. Remote Preconditioning (RPC)

RPC is a biological mechanism of interorgan protection against IR injury [170, 171]. Brief cycles of IR applied to a tissue remote from the heart, such as the small intestine [172] or the upper or lower limb [173], before the onset of myocardial ischemia limit myocardial infarct size. A comparison of RPC and IPC induced by occlusion of the superior mesenteric artery and the left coronary artery, respectively, in a rat model of myocardial IR injury showed a greater effect of IPC compared with RPC in terms of infarct size reduction [174]. In this study, IPC was associated with increased ERK1/2 and JNK1 activation but reduced p38 MAPK activation in the heart. In contrast, RPC triggered by occlusion of the superior mesenteric artery induced ERK1/2 and JNK1 activation in the small intestine without participation of MAPKs in the heart. Each of the applied ERK1/2, JNK, and p38 MAPK inhibitors abrogated RPC-mediated protection. An underlying mechanism may be PKC*ε* isoform activation in the heart via remote ischemia-induced transmitter release [175]. A distinct study showed equivalent degrees of cardioprotection induced by IPC and RPC, while suggesting a role for bradykinin as a mediator of cardiac PC at a distance [176].

## 6. Postconditioning (PostC)

Ischemic PostC can be elicited by repetitive cycles of rapid reflow/reocclusion in the initial 2 min after release of a protracted coronary occlusion [29, 30, 177–181]. Because tissue injury is initiated within minutes of reperfusion, PostC must be applied at the onset of reperfusion [181]. PostC has limited infarct size in all species tested so far [177, 178, 182–184], including humans [185, 186]. The degree of PostC-mediated cardioprotection is comparable to that induced by IPC [177, 178, 186], or slightly lower than it [187]. PostC activates adenosine receptors and the NO/cGMP pathway [188, 189], mitochondrial K_ATP_ channels, PKC and protein kinase G (PKG) [190], and the RISK pathway including ERK1/2 [188] and PI3K/Akt [184, 189, 191]. In the rabbit model of myocardial IR injury, an ERK1/2 inhibitor abolished protection by brief episodes of coronary occlusion applied at reperfusion [188]. PostC has also been shown to reduce oxidative stress in a senescent mouse model [192] and to attenuate cardiomyocyte apoptosis after simulated ischemia via JNK and p38 MAPK inhibition [193]. Moreover, PostC has been shown to inhibit MPTP opening in the early minutes of reperfusion [194]. 

The RISK pathway is not the only cardioprotective pathway [195]. In mouse and rabbit hearts, protection after ischemic PostC was associated with increased activation of ERK, but not Akt [183, 196]. In pigs, ischemic PostC enhanced ERK and Akt activation during reperfusion without a decrease in infarct size [197]. A distinct study in anesthetized pigs demonstrated myocardial protection after PostC without an increase in Akt, ERK, and GSK-3*β* phosphorylation and with no effect of PI3K or ERK1/2 blockade [198]. Gentle reperfusion likewise reduced infarct size in pigs without activation of the RISK pathway [199]. The so-called “Survivor Activating Factor Enhancement” (SAFE) pathway [200] which includes the JAK-STAT signaling pathway [94, 95], may be responsible for cardioprotection in the absence of activation of the RISK pathway.

Pharmacological stimuli including inhalational anesthetics can replace the ischemic PostC stimulus applied at the onset of reperfusion [201–203]. While myocardial protection after ischemic PostC is not enhanced by IPC [187], pharmacological PostC and IPC or pharmacological PC may have additive effects.

## 7. IPC, RPC, and PostC for Protection against Myocardial IR Injury in Humans

Recently, IPC, RPC, and PostC strategies for attenuating myocardial IR injury have been tested in clinical trials in nontransplanted patients [77]. Both IPC and pharmacological PC reduced myocardial IR injury in patients undergoing coronary artery bypass graft surgery [204–207]. In a randomized controlled trial, RPC triggered by a simple noninvasive technique of four 5 min cycles of lower limb ischemia and reperfusion induced cardioprotection in children undergoing cardiac surgery for congenital heart disease [208]. In a distinct randomized controlled trial, RPC triggered by transient upper limb ischemia induced cardioprotection in adult patients undergoing coronary artery bypass graft surgery [209]. In the prospective randomized controlled cardiac remote ischemic preconditioning in coronary stenting (CRISP Stent) trial, RPC alleviated ischemic chest discomfort and myocardial injury during coronary stenting, while also reducing subsequent cardiovascular events [210]. In a randomised trial in patients with acute myocardial infarction undergoing angioplasty, ischemic RPC before hospital admission proved to be safe and appeared to salvage ischemic myocardium [211]. 

Ischemic PostC has been evaluated in patients with ST elevation myocardial infarction (STEMI) undergoing angioplasty [185]. Within the first minute after stent implantation, patients in the PostC group underwent four cycles of 1 min inflation and 1 min deflation of the coronary angioplasty balloon. Creatine kinase release, measured as a surrogate for infarct size, was significantly reduced by 36% in PostC versus control patients. Contractile function was still improved in the PostC group at 1 year following infarct [212]. Whether or not PostC protects against endothelial IR injury in humans remains unclear [213, 214]. 

To our knowledge, no data on IPC, RPC, or PostC in human heart transplantation have been published so far. Analogously, data on MAPK inhibitors in this setting are restricted to animal models, as discussed in the next section.

## 8. MAPK Inhibition in Experimental Heart Transplantation

ERK1/2, JNK, and p38 MAPK activation within cardiac grafts has been demonstrated in dogs [215]. MAPK activation can contribute to graft injury via multiple mechanisms including cytokine upregulation [216–219], immune cell activation, and apoptosis.

JNK promotes T-cell activation and differentiation. For instance, JNK and ERK1/2 have been shown to stimulate IL-2 production by Thy-1-activated mouse T lymphocytes in vitro [220]. JNK inhibition reduced histological rejection and improved graft survival in a rat model of heart transplantation [221]. 

p38 MAPK is involved in IL-2R signaling in T lymphocytes, while also stimulating cytokine release from human macrophages in vitro [222]. A p38 MAPK inhibitor administered at reperfusion improved functional recovery of rat hearts after prolonged hypothermic ischemia [223]. In a brain-dead donor model, a p38 MAPK inhibitor lowered systemic levels of proinflammatory cytokines while not affecting intracardiac cytokine levels [224]. Addition of a p38 MAPK inhibitor to the Celsior solution enhanced the viability of cardiac grafts from non-heart-beating donors in a canine model of heart transplantation [225]. Moreover, p38 MAPK blockade attenuated the release of proinflammatory IL-6 by human endothelial cells in vitro after cooling and rewarming [226]. p38 MAPK inhibition similarly prevented endothelial adhesion molecule expression and polymorphonuclear accumulation after myocardial IR injury in rats [74]. p38 MAPK blockade markedly reduced vascular smooth muscle cell proliferation in aortic grafts and the development of graft vasculopathy [227]. Finally, addition of a p38 MAPK inhibitor to the Euro-Collins and University of Wisconsin solutions mitigated IR injury in lung [228] and liver [229] grafts, respectively, as well as in kidney grafts from non-heart-beating donors [230]. Thus, a p38 MAPK inhibitor applied during organ procurement and storage can protect the graft against IR injury.

## 9. PC and PostC in Experimental Heart Transplantation

The potential relevance of PC and PostC strategies to organ transplantation has been reviewed elsewhere [231–233]. Proof-of-principle studies in animal models have demonstrated that IPC can impart protection on cardiac grafts [234–236]. Pretreatment of rat hearts with an adenosine analog prior to harvesting and storage in the Euro-Collins solution for 8 hours improved functional recovery at reperfusion [237]. In another study, IPC combined with Na^+^/H^+^ antiporter inhibition improved cardiac function in rat hearts after 4 hours of storage at 4°C in Celsior solution and extracorporeal reperfusion [238]. K_ATP_ channel activation mimicked the protective effect of IPC in hearts after prolonged hypothermic storage [239–241]. However, one study showed IPC-induced cardioprotection after global ischemia, but not after cold cardioplegia [242]. Also, brain death completely abolished PC-mediated protection in ischemic rabbit hearts [243]. This finding might be explained by catecholamine storm after brain death, since norepinephrine injection before IPC abolished protection in the absence of brain death [244]. AMP-activated protein kinase (AMPK) is emerging as a target for PC in transplantation medicine [245].

PC induced by sildenafil administration to the donor 30 min before the onset of ischemia improved the function of cardiac grafts after 3 h of hypothermic cardioplegic arrest [246]. In contrast, PostC induced by sildenafil administration 5 min before reperfusion in the recipient was ineffective.

PKC*δ* inhibition improved cardiac contractile performance and coronary perfusion after cold cardioplegic arrest in isolated rat hearts [247]. This approach similarly attenuated heart transplant injury and graft coronary vasculopathy after prolonged organ ischemia [248]. Isoflurane as well as inhaled hydrogen or carbon monoxide has been shown to alter energy substrate metabolism to preserve mechanical function in isolated rat hearts after extended no-flow hypothermic storage [249, 250]. 

Ischemic RPC was tested in a pig model of orthotopic heart transplantation from brain-dead donors [251]. RPC of the recipient by four 5 min cycles of lower limb ischemia attenuated IR injury of the denervated donor heart via a K_ATP_ channel-dependent mechanism.

Ischemic PostC was tested in isolated working rat hearts after global total ischemia (4 h/4°C) and 45 min of reperfusion [252]. Three brief episodes of total global ischemia applied at the onset of reperfusion reduced myocardial injury and postischemic dysfunction. In another study, both PostC and remote PostC attenuated tissue damage in warm ischemic rat cardiac grafts [253].

The first clinical application of IPC in solid organ transplantation concerned liver transplantation [254]. Although IPC mitigated inflammatory responses [255], it was associated with initial poor function. It did neither improve nor compromise the outcome of cadaver liver transplantation [254]. 

## 10. Concluding Remarks and Perspectives

Proof-of-principle studies have provided evidence that therapeutic manipulation of the donor heart at the time of transplantation can mitigate graft injury, immunogenicity, and rejection. A possibility is that molecular events during the triggering phase of PC, which induce protection, can be applied to the donor heart before transplantation. A preconditioning drug (e.g., sildenafil) can be administered to the donor before organ retrieval and/or 5 min before reperfusion in the recipient [246]. The clinical efficacy of ischemic PostC in STEMI patients [185] suggests that this approach might be beneficial in heart-transplanted patients as well. A p38 MAPK inhibitor can be added to an organ preservation solution or administered at reperfusion [223, 225]. A p38 MAPK inhibitor administered to the recipient markedly inhibited the development of aortic graft vasculopathy in an experimental model [227]. Small-molecule inhibitors of p38 MAPK have been developed [256] and tested in initial clinical trials in patients with active rheumatoid arthritis or neuropathic pain [257, 258]. Further preclinical studies are needed, however, before these drugs can be tested in heart transplant recipients. In principle, extended p38 MAPK inhibitor administration during several weeks or months after transplantation might protect against graft vasculopathy.

Because distinct MAPK isoforms have different substrate affinities and functions [61, 145, 159], the precise identification of MAPK isoforms that contribute to IR injury would allow for the development of targeted therapies. Avoiding indiscriminate MAPK blockade is important because MAPK activates signaling pathways participating in host defense against infection and tumors.

Despite promising results obtained with MAPK inhibitors as well as PC and PostC in animal models, it should be noted that clinical trials of cardioprotective agents successfully tested in animal models have been largely negative so far [259]. However, a recent trial suggested a protective effect of cyclosporine, a MPTP opening inhibitor, against reperfusion injury in patients with acute myocardial infarction [260]. In transplantation medicine, MAPK inhibitors will need to be tested in combination with other PC and PostC strategies, as well as with improved organ preservation solutions and reperfusion protocols (e.g., continuous myocardial perfusion and controlled initial reperfusion) [261–263].
